# MiR-32-5p influences high glucose-induced cardiac fibroblast proliferation and phenotypic alteration by inhibiting DUSP1

**DOI:** 10.1186/s12867-019-0135-x

**Published:** 2019-08-22

**Authors:** Jie Shen, Wanhong Xing, Rui Liu, Yiying Zhang, Chunhong Xie, Fangqi Gong

**Affiliations:** 1 Department of Cardiology, Children’s Hospital, Zhejiang University School of Medicine, No. 3333 Binsheng Road, Binjiang District, Hangzhou, 310051 Zhejiang China; 2 Children’s Heart Center, Sichuan Provincial Hospital for Women and Children, Chengdu, 610045 Sichuan China; 3 Department of Physiology and Pathophysiology, School of Basic Medical Science, Xi’an Jiaotong University Health Science Center, Xi’an, 710061 Shaanxi China

**Keywords:** Cardiac fibroblast, High glucose, miR-32-5p, DUSP1

## Abstract

**Background:**

The current study aimed to investigate the effects of miR-32-5p on cardiac fibroblasts (CFs) that were induced with high levels of glucose; we also aimed to identify the potential mechanisms involved in the regulation of DUSP1 expression.

**Methods:**

Human CFs were transfected with a miR-32-5p inhibitor or mimic and were treated with a normal concentration or a high concentration of glucose. Flow cytometry analysis was performed to identify cardiac fibroblasts by examining vimentin, fibronectin (FN) and α-actin expression in human CFs. qRT-PCR and western blot assays were performed to confirm the expression of miR-32-5p, DUSP1 and cardiac fibrosis relevant proteins. The proliferation of CFs was assessed by using MTT assay. An immunocytofluorescent staining assay was performed to determine the protein level of α-SMA and to investigate the degree of phenotypic changes in human CFs. The specific relationship between miR-32-5p and DUSP1 was investigated by a dual luciferase reporter assay. Cell apoptosis rates were measured with flow cytometry and the annexin V-FITC and propidine iodide (PI) staining method.

**Results:**

A luciferase reporter assay indicated that miR-32-5p could directly target DUSP1. High glucose levels resulted in the overexpression of miR-32-5p, which downregulated DUSP1 expression. Both the upregulation of miR-32-5p and the downregulation of DUSP1 promoted cell apoptosis, proliferation and phenotypic changes in human CFs.

**Conclusions:**

All findings in this study provide further evidence for the positive effects of miR-32-5p on cell proliferation and the phenotypic changes in CFs by inhibiting DUSP1 expression, and reveal that miR-32-5p could serve as prognostic diagnostic target for cardiac fibrosis.

**Electronic supplementary material:**

The online version of this article (10.1186/s12867-019-0135-x) contains supplementary material, which is available to authorized users.

## Background

Diabetes mellitus (DM) is a metabolic disorder and a major health concern in worldwide [1]. Chronic DM can result in the generation of cardiovascular complications including heart failure, myocardial fibrosis, and cardiac dysfunction [2]. Cardiac fibrosis is considered to be a major pathogenic component of many cardiovascular diseases by impairing pumping capacity and increasing myocardial stiffness; these changes ultimately results in fatal arrhythmia [3, 4]. However, so far there is no curative treatment for cardiac fibrosis.

Cardiac fibroblasts (CFs) are the main cellular constituents of the heart and account for 27% of the total myocardial mass [5], CFs play major roles in the synthesis and degradation of extracellular matrix (ECM) proteins, which regulate the maintenance and development of functional heart tissue. [6]. This study analyzed the expression of transforming growth factor-β (TGF-β), collagen I, and collagen III in CFs. Collagens are major ECM proteins, TGF-β is a key factor in the regulation of ECM proteins expression, and the overexpression of TGF-β can result in tissue fibrosis [7]. High glucose can induce collagen synthesis and the proliferation of cardiac fibroblasts and vascular smooth muscle cells, which could further result in the cardiovascular complication of diabetes [8, 9]. The pathophysiological changes of CFs are closely associated with the generation of cardiac fibrosis, and the increased collagens deposition might eventually contribute to more severe fibrosis [10, 11]. Some previous studies have identified that high glucose (HG) or hyperglycemia is able to induce collagen synthesis and promote the myofibroblast differentiation in vitro [12–14]. However, the underlying mechanisms of high glucose-induced cardiac fibrosis are still unclear.

MiRNAs are a group of small noncoding RNAs that are approximately 22 nucleotides long, and these miRNAs can bind to the 3′UTR of a targeted mRNA with partially complementary sequences and can regulate the expression of the target mRNA [15]. MiRNAs have been implicated in many human diseases. Previous studies have reported the effects of miRNAs on suppressing mRNA translation and degrading mRNA [16]. As a result, development, proliferation, apoptosis, and other necessary cellular process are affected. Additionally, some recent studies have determined that the development and progression of cardiac fibrosis is related to the dysregulation of miRNAs. The overexpression of miR-19b [17] and miR-125b [18] was demonstrated to induce the progression of cardiac fibrosis, while the upregulation of miR-9 [19] had the opposite effect. Moreover, numerous miRNAs have been verified to be potential regulators for the expression of genes involved in glucose stress [20].

Dual-specificity protein phosphatase (DUSP) is known as mitogen-activated protein kinase (MAPK) phosphatase [21]. High DUSP1 expression can be found in several tissues, including the heart, lungs, and liver [21]. In the heart, DUSP1 regulates cardiac metabolism, and the inhibition of DUSP1 (or MKP1) was reported to induce cardiac hypertrophy in a transgenic mouse model [22]. In addition, a study by Weng et al. reported that the expression of DUSP1 was low in the myocardium of a diabetic rat; this suggests that the abnormal expression of DUSP1 might involve the pathophysiology of diabetic cardiomyopathy [23].

In the present study, we hypothesized that the down-regulation of miR-32-5p might have an effect on the TGF-β_1_ expression, collagen accumulation, and HG-induced proliferation of human CFs. Besides, we analyzed the target relationship between miR-32-5p and DUSP1, to determine whether the regulation of DUSP1 in human CFs could be affected by the overexpression or inhibition of miR-32-5p. Moreover, the influence of miR-32-5p on the proliferation and phenotypic alteration of human CFs were investigated, suggesting a novel therapeutic approach on cardiac fibrosis.

## Results

### The identification of CFs

**Fig. 1:**
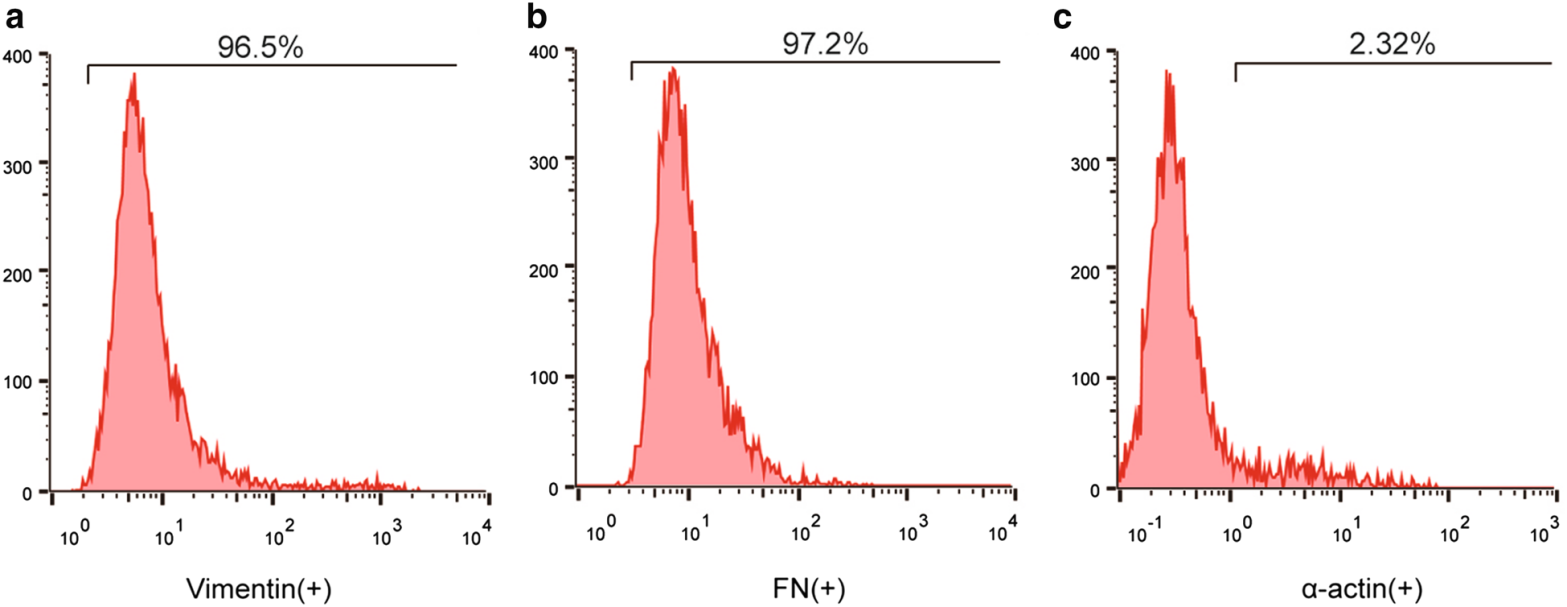
The identification of cardiac fibroblasts. **a** Flow cytometry analysis of vimentin expression in human cardiac fibroblasts (CFs). **b** Flow cytometry analysis of fibronectin (FN) expression in human CFs. **c** Flow cytometry analysis of α-actin expression in human CFs

CFs were identified through flow cytometry analysis. Fibroblast-specific markers: vimentin and fibronectin were detected. Vimentin is a type III intermediate filament protein in interstitial cells, and the intermediate filaments are a part of the cytoskeleton. Vimentin-positive expression was presented in CFs. Fibronectin is a protein that is present in fibroblasts [24]. Specifically, CFs were negative for α-actin [25]. Based on the results of Fig. 1, CFs were observed to have significant populations that were positive for vimentin (96.5%) and fibronectin (97.2%), and were negative for α-actin (2.32%) (Fig. 1a–c). The data indicate that our samples are highly purified CFs.

### High glucose-induced CFs had increased levels of miR-32-5p, apoptosis and the proliferation rate

**Fig. 2:**
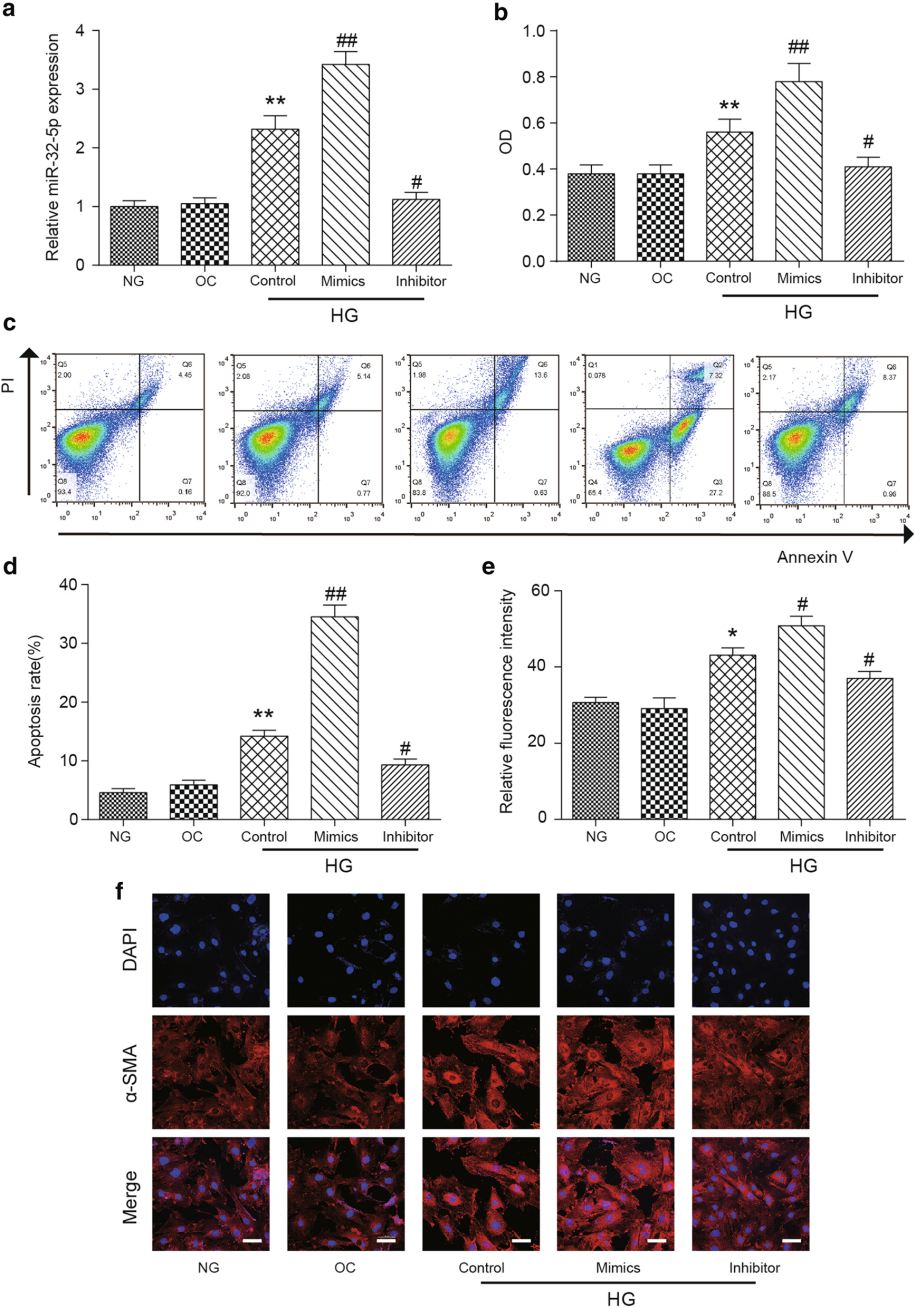
Downregulated miR-32-5p in human CFs. **a** The expression level of miR-32-5p in the normal glucose (5.6 mM), osmotic control (5.6 mM glucose and 19.4 mM mannitol), and high glucose groups (25 mM). The mimic group was treated with miR-32-5p mimics for 48 h, and the inhibitor group was treated with a miR-32-5p inhibitor for 48 h. **b** The OD_595_ values of human CFs cultures in different experimental groups were determined by an MTT assay. **c**, **d** The results of an apoptosis assay in different experimental groups are shown. **e**, **f** The α-SMA expression of CFs, which was detected by a double-label immunofluorescence-staining technique, is shown red fluorescent cells indicate that α-SMA was expressed in CFs, Scale bar = 50 μm. The data are presented as the mean ± SEM (n = 3). *p < 0.05, **p < 0.01 (compared with the NG group), ^#^*p *< 0.05, ^##^*p *< 0.01 (compared with the HG Control group)

To determine whether the expression of miR-32-5p was related to the high glucose induced cardiac fibrosis in vitro, qRT-PCR was used in this study to analyze the expression level of miR-32-5p in human CFs incubated with normal (5.6 mM) and high (25 mM) concentrations of glucose for 48 h. The results in Fig. 2a show that the miR-32-5p expression in the control HG group was significantly higher than that in the NG group (normal glucose, *p *< 0.01). For cells treated with 5.6 mM glucose and 19.4 mM mannitol, the miR-32-5p expression level had no significant difference compared to that in the NG group (Fig. 2a). In terms of the miR-32-5p expression in transfected CFs treated with high glucose, miR-32-5p was highly expressed in the high glucose miR-32-5p mimics group (*p *< 0.01), while a significantly lower expression level of miR-32-5p was observed in the high glucose inhibitor group (*p *< 0.05) compared to that in the HG Control group (Fig. 2a). To verify the effects of different concentration of glucose on miR-32-5p or DUSP1, the expression of miR-32-5p and DUSP1 were detected under different concentration of glucose in CFs. The results showed that the expression of miR-32-5p was increased and the expression of DUSP1 was decreased with the increase of glucose concentration (Additional file 1: Figure S1). The high glucose induced cell cultures observed significantly higher OD (optical density) values compared to that in the NG group, indicating that high glucose levels promoted cell proliferation (*p *< 0.01, Fig. 2b). For cell cultures with high concentration of glucose, the OD value in the miR-32-5p mimics group was significantly increased (*p *< 0.01), while the OD value in the miR-32-5p inhibitor group was decreased (*p *< 0.05) compared to those in the control HG group (Fig. 2b).

The apoptosis rate of CFs was detected by using the flow cytometry method. The results indicated that no variation of the apoptosis rate was observed between the NG and OC groups. The apoptosis rate in the high glucose cell cultures was increased significantly compared with that in the NG group (*p *< 0.01). For the transfected human CFs, the apoptosis rate in the mimics group was strongly increased compared to that in the HG Control group (*p *< 0.01), while the apoptosis rate in the inhibitor group was decreased compared to that in the HG Control group (*p *< 0.05) (Fig. 2c, d).

In conclusion, the expression of miR-32-5p was upregulated in CFs, its overexpression could promote the apoptosis rate of human CFs.

### The detection of α-SMA in human CFs

To identify whether CFs change from fibroblasts to myofibroblasts under different conditions, a double-label immunofluorescence staining assay was performed to measure the expression level of α-SMA in CFs. Alpha-smooth muscle actin (α-SMA) is a marker of myofibroblast phenotypes [26]. There was no variance observed in the relative fluorescence intensity between the OC group and the NG group, while the relative fluorescence intensity in the HG Control group was significantly higher compared to those in the NG group and the OC group (*p *< 0.01). Moreover, the fluorescence intensity was significantly increased in the mimics group (*p *< 0.01) and was significantly decreased in the inhibitor group (*p *< 0.05) compared with that in the HG Control group (Fig. 2e). Besides, α-SMA-positive cells with red fluorescence indicated that α-SMA was expressed in human CFs (Fig. 2f). Stronger red fluorescence was observed in the HG Control group compared to that in the NG groups, suggesting that high glucose might be able to induce a phenotypic change.

### MiR-32-5p induced the expression of TGF-β_2_, collagen I, and collagen III in CFs

**Fig. 3:**
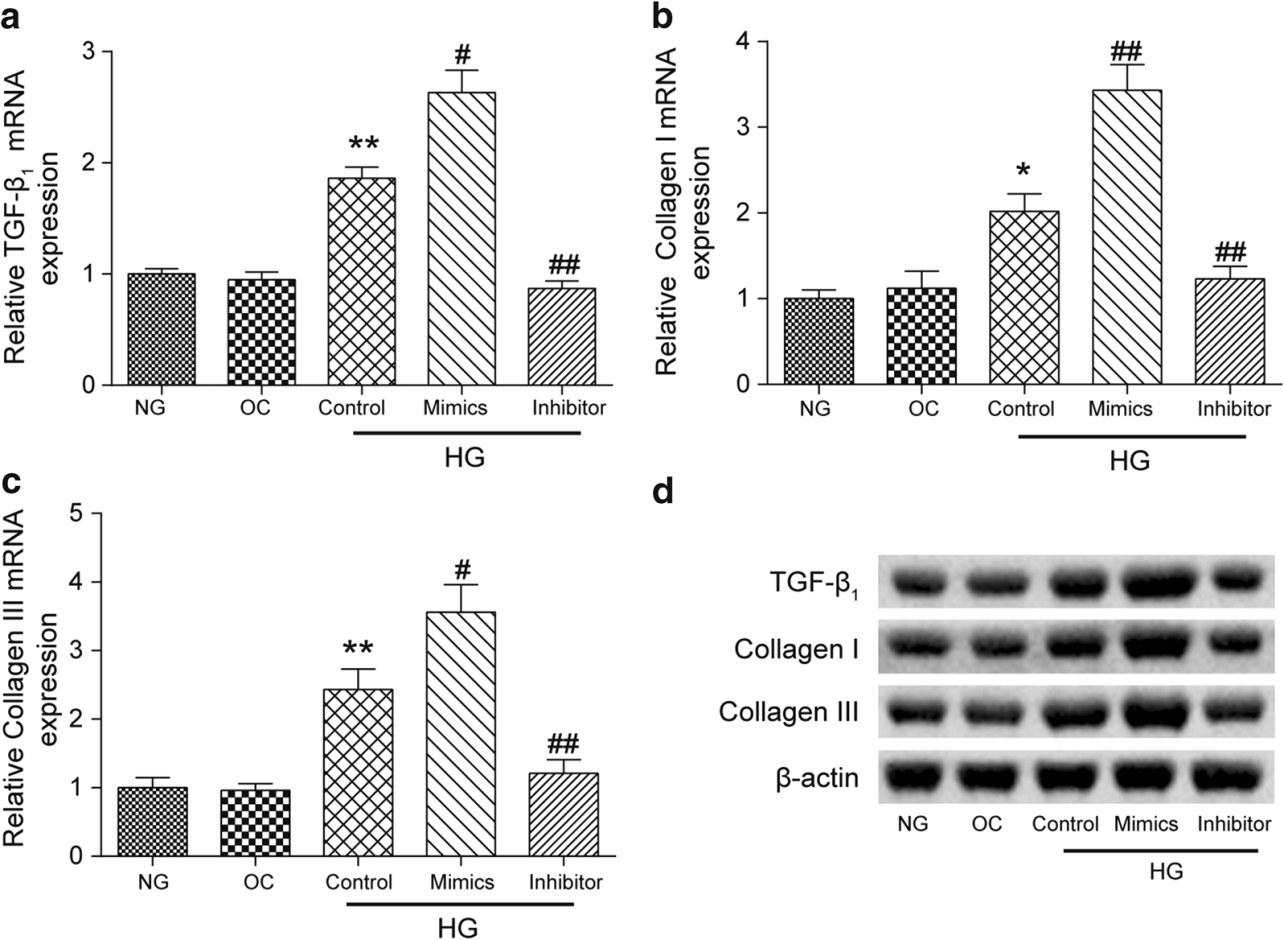
Expressions of TGF-β_1_, collagen I, and collagen III at the mRNA and protein levels in different experimental groups. The mRNA levels of **a** TGF-β_1_, **b** collagen I, and **c** collagen III were quantified by a qRT-PCR analysis. **d** The protein levels of TGF-β_1_, collagen I, and collagen III were determined by a western blot assay. β-actin was used as a loading control. The data were presented as the mean ± SEM (n = 3). *p < 0.05, **p < 0.01 (compared with the NG group), ^#^*p *< 0.05, ^##^*p *< 0.01 (compared with the HG Control group)

The mRNA and protein expression levels of three cardiac fibrosis relative proteins, TGF-β_1_, collagen I, and collagen III, were measured by RT-PCR and western blot assays, respectively. There was no significant difference in the expression of these three proteins between the NG and OC groups. When cultured with high concentration of glucose, the expression levels of TGF-β_1_ (*p *< 0.01), collagen I (*p *< 0.05), and collagen III (*p *< 0.01) in cell cultures were increased significantly compared to those in the NG group. Further, for cells cultured in the miR-32-5p mimics group, high expression levels of TGF-β_1_, collagen I and collagen III were observed, while these three relative proteins showed significantly lower expression levels in the miR-32-5p inhibitor group compared with those in the HG Control group (*p *< 0.01, Fig. 3a–c).

All the results obtained from qRT-PCR were confirmed by a western blot assay and are shown in Fig. 3d. The expression of β-actin served as the loading control.

### MiR-32-5p directly targeted DUSP1 in human CFs

**Fig. 4:**
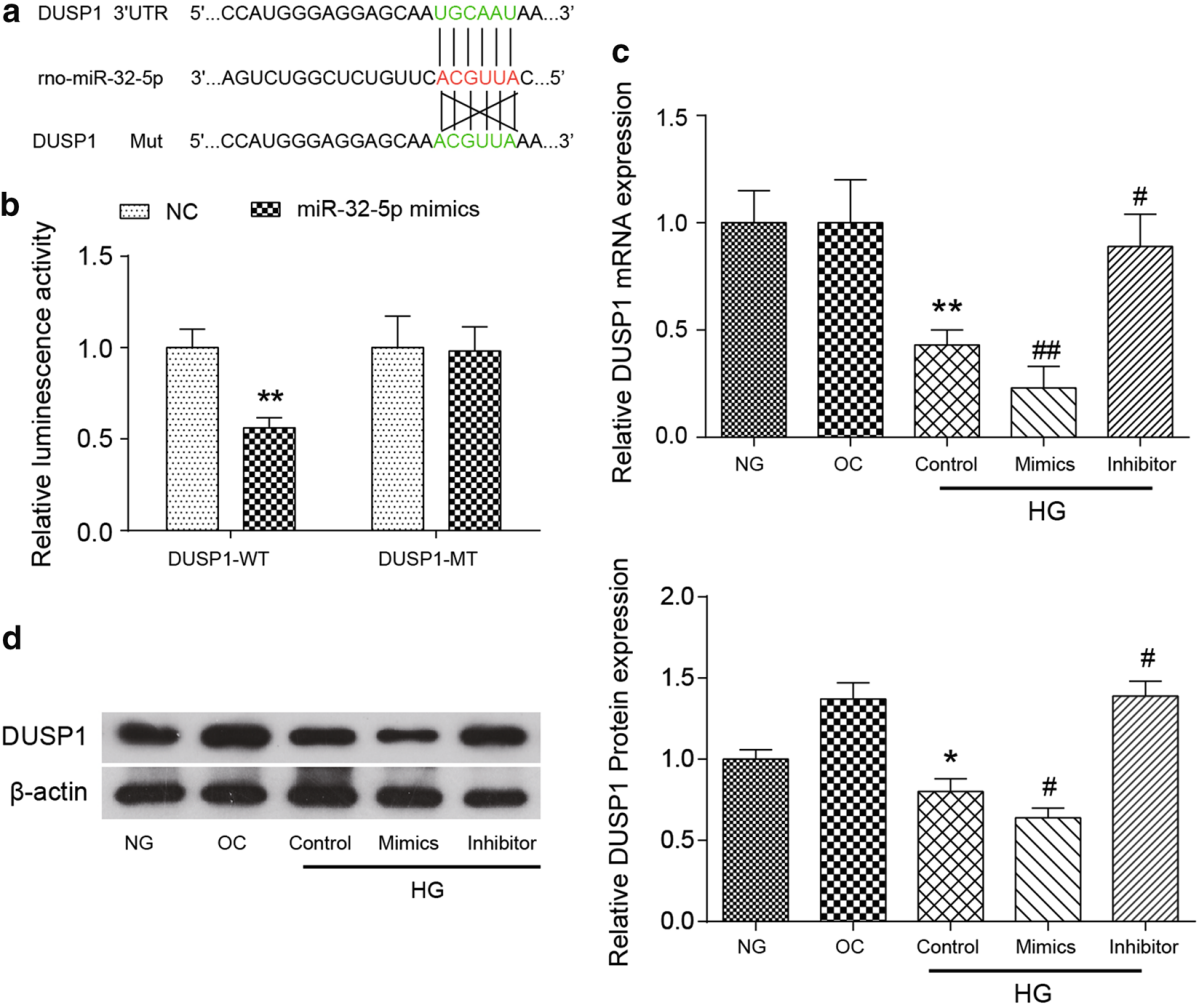
DUSP1 was a direct target of miR-32-5p. Human CFs were transfected with a miR-32-5p inhibitor or mimic for 48 h. **a** A schematic representation of the DUSP1 3′UTR showing putative miRNA target sites is shown. **b** The analysis of the relative luminescence activities of DUSP1-WT and DUSP1-MT in human CFs is shown. The luminescence activity of DUSP-1 WT + mimics was relative to that of the DUSP1-WT NC group, and the luminescence activity of DUSP-1 MT + mimics was relative to that of the DUSP1-MT NC group. **c** The mRNA level of DUSP1 expression was quantified by a qRT-PCR analysis in the CFs of each experimental group. **d** The protein levels of DUSP1 were determined by a western blot assay in the CFs of each experimental group. β-actin was used as a loading control in western blot. The data are presented as the mean ± SEM (n = 3). *p < 0.05, **p < 0.01 (compared with the NG group or the NC group), ^#^*p *< 0.05, ^##^*p *< 0.01 (compared with the HG Control group)

The miR-32-5p binding site in the 3′UTR of DUSP1 has been identified through the database TargetScan 6.2 (Fig. 4a). To demonstrate the direct binding relationship between miR-32-5p and DUSP1, luciferase vectors containing the potentially-targeted DUSP1 3′UTR region (WT) or a mutated DUSP1 3′UTR were constructed, and the luminescent activity was detected (Fig. 4b). We observed that the overexpression of miR-32-5p inhibited the wild-type DUSP1 reporter activity, while no change was observed in the luminescent activity of the mutated DUSP1 reporter, suggesting that miR-32-5p could specifically target the DUSP1 3′UTR through binding to the seed sequence.

The mRNA and protein levels of DUSP1 were confirmed by qRT-PCR and western blot assays respectively. We found that the high concentration of glucose could significantly decrease the expression of DUSP1 mRNA and the expression of DUSP1 protein, compared with those of the NG group (*p *< 0.01). In addition, the introduction of miR-32-5p could further suppress the DUSP1 mRNA (*p *< 0.01) and protein expression (*p *< 0.05) compared to those in the HG Control group, while inhibiting miR-32-5p could increase the expression of both the DUSP1 mRNA and protein levels compared to those of the HG Control group (*p *< 0.05, Fig. 4c, d). The expression of β-actin served as the loading control in western blot assay. All the results of the qRT-PCR and western blot assays indicated that DUSP1 was downregulated in high glucose-induced CFs. MiR-32-5p can negatively regulate the expression of DUSP1 via directly binding to the 3′UTR of DUSP1.

### MiR-32-5p promoted the apoptosis and proliferation of human CFs by inhibiting DUSP1 expression

**Fig. 5:**
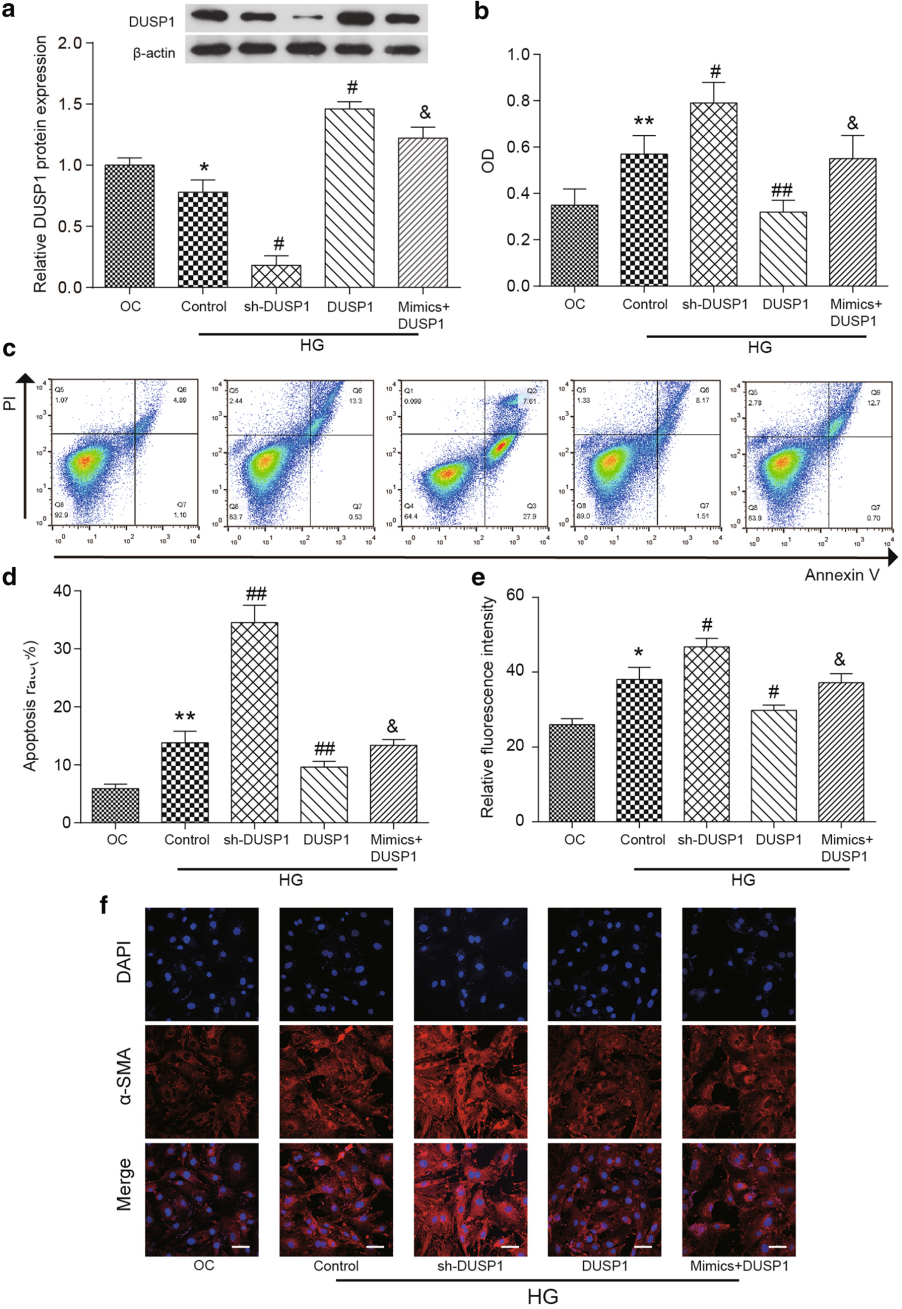
Upregulated DUSP1 in human CFs. **a** The expression level of DUSP1 protein in different experimental groups was determined by a western blot assay. β-actin was used as a loading control. **b** The OD_595_ values of human CFs cultures in different experimental groups were determined by an MTT assay. **c**, **d** The results of an apoptosis assay in different experimental groups. **e**, **f** The α-SMA expression of CFs was detected by a double-label immunofluorescence-staining technique. Red fluorescent cells indicate that α-SMA was expressed in CFs, Scale bar = 50 μm. The data are presented as the mean ± SEM (n = 3). *p < 0.01, **p < 0.01 (compared with the OC group), ^#^*p *< 0.05, ^##^*p *< 0.01 (compared with the HG Control group), ^&^*p *< 0.05, (compared with the DUSP1 group)

Since the target relationship between miR-32-5p and DUSP1 has been identified, it is necessary to further investigate how the miR-32-5p/DUSP1 axis regulates the apoptosis rate and the proliferation rate of human CFs. We observed that DUSP1 expression was reduced remarkedly in the HG Control group compared with that in the OC group (*p *< 0.01), which suggests that the high concentration of glucose might suppress the expression of DUSP1. The expression level of DUSP1 was decreased significantly in the sh-DUSP1 group, and was increased significantly in the DUSP1 group (overexpressed DUSP1) compared to that of the HG Control group (*p *< 0.01), this confirmed the successful transfection of human CFs. However, the presence of overexpressed miR-32-5p in the DUSP1 cell cultures (mimics + DUSP1 group) strongly repressed the DUSP1 expression level compared to that in the DUSP1 group (*p *< 0.01, Fig. 5a); again, this demonstrates that the targeting function of miR-32-5p to DUSP1 could suppress DUSP1 expression in CFs. All the results were confirmed by western blot assay.

In terms of cell proliferation, it was found that the OD value of cells was significantly higher in the sh-DUSP1 group (*p *< 0.05) and was significantly lower in the DUSP1 group (*p *< 0.01) than that in the HG Control group. In addition, overexpressing miR-32-5p in the mimics + DUSP1 group significantly reversed the decreased OD value in the DUSP1 group (*p *< 0.05, Fig. 5b).

The flow cytometry assay was performed to measure the apoptosis rate of human CFs. The apoptosis rate of cells in the HG Control group was significantly increased compared to that of the OC group (*p *< 0.01). Moreover, the apoptosis rate of CFs was increased significantly in the sh-DUSP1 group and was dramatically decreased in the DUSP1 group compared to that of the HG Control group (*p *< 0.01). Similarly, the presence of overexpressed miR-32-5p in the mimics + DUSP1 group could reverse the decrease in the apoptosis rate in the DUSP1 group (*p *< 0.05) (Fig. 5d).

### MiR-32-5p promoted α-SMA expression in human CFs by inhibiting DUSP1 expression

To further investigate how the miR-32-5p/DUSP1 axis regulates the phenotypic change of human CFs, the expression level of α-SMA was determined again with a double-label immunofluorescence staining assay. The results in Fig. 5e, f showed that the fluorescence intensity in the HG group was significantly higher than that of the OC group (*p *< 0.01). For the high glucose-induced human CFs, the fluorescence intensity was increased in the sh-DUSP1 group and was decreased in the DUSP1 group compared to that in the HG Control group (*p *< 0.01). Compared to that in the DUSP1 group, the fluorescence intensity was increased significantly in the mimics + DUSP1 group (*p *< 0.05). The results demonstrated that miR-32-5p could suppress the effect of DUSP1 on suppressing the phenotypic alteration.

### The inhibition effect of DUSP1 on the expression of TGF-β_1_, collagen I, and collagen III was suppressed by miR-32-5p

**Fig. 6:**
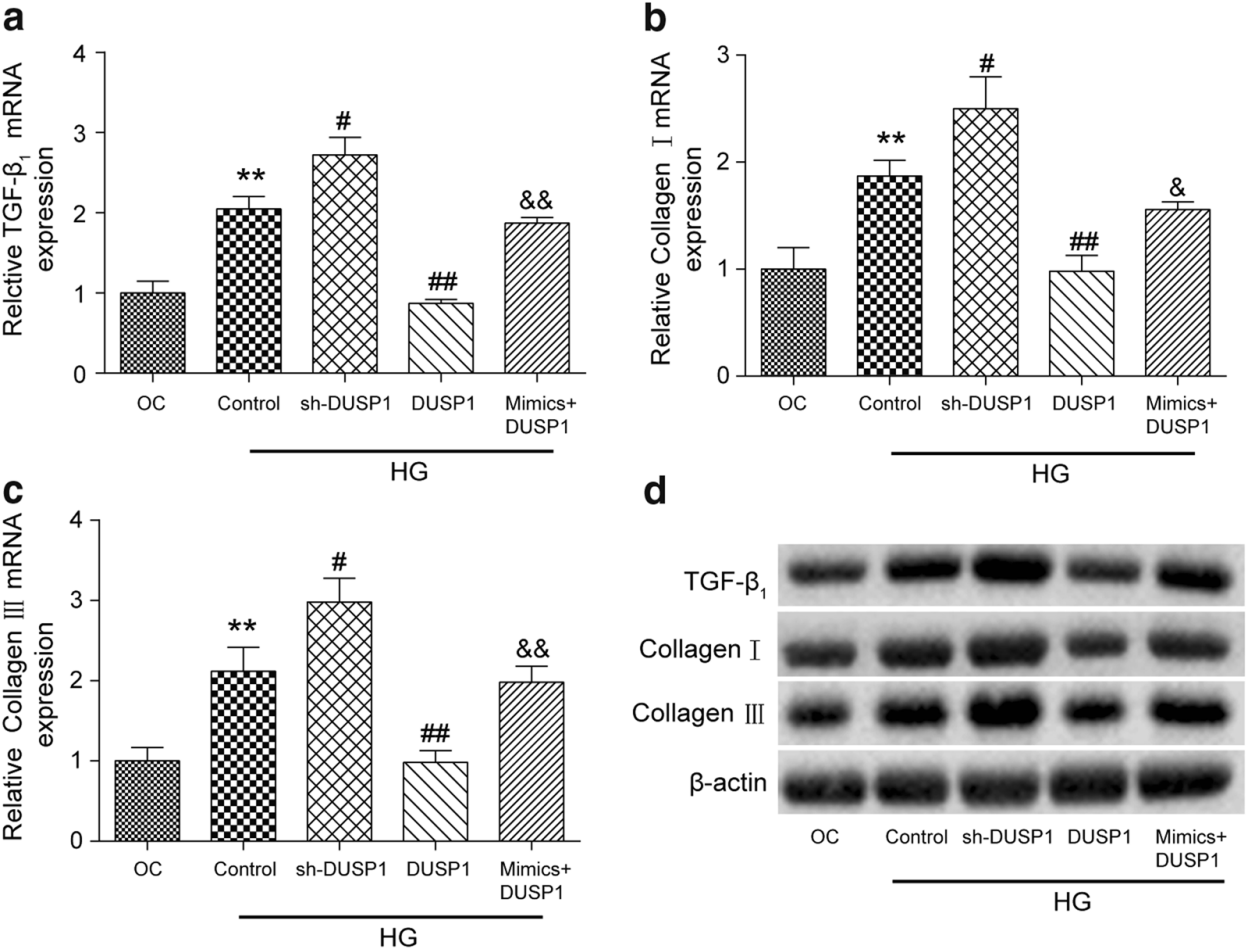
Expressions levels of TGF-β_1_, collagen I, and collagen III at the mRNA and protein levels in different experimental groups. The mRNA levels of **a** TGF-β_1_, **b** collagen I, and **c** collagen III were quantified by a qRT-PCR analysis in the CFs of each experimental group. **d** The protein levels of TGF-β_1_, collagen I, and collagen III were determined by a western blot assay in CFs of each experimental group. β-actin was used as a loading control. The data are presented as the mean ± SEM (n = 3). **p < 0.01 (compared with the OC group), ^#^*p *< 0.05, ^##^*p *< 0.01 (compared with the HG Control group), ^&^*p *< 0.05, ^&&^*p *< 0.01 (compared with the DUSP1 group)

The changes of the expression of TGF-β_1_, collagen I, and collagen III at the mRNA and protein levels were detected by qRT-PCR and western blot analyses. As expected, the mRNA expression levels of these three proteins were increased significantly in the HG Control group compared to that of the OC group (p < 0.01, Fig. 6a–c). The inhibition of DUSP1 in human CFs increased the protein expression levels (p < 0.05) while the overexpression of DUSP1 resulted in a significant decrease in the expression levels of three cardiac fibrosis-related proteins (p < 0.01) compared to those of the HG Control group. Moreover, the mimics + DUSP1 cotransfection group observed increased TGF-β_1_ (p < 0.01), collagen I (p < 0.05), and collagen III expression levels (p < 0.01) compared to those of the DUSP1 group.

All the results obtained from qRT-PCR were confirmed by a western blot assay and are shown in Fig. 6d. The expression of β-actin served as the loading control.

## Discussion

In the current study, miR-32-5p was found to suppress DUSP1 expression in human CFs. High glucose increased the expression of miR-32-5p, which was found to promote cell apoptosis, enhance cell proliferation and induce a phenotypic alteration in human CFs. In contrast, DUSP1 was observed to be downregulated in high glucose-treated human CFs. Our study indicates that the downregulation of DUSP1 had similar impacts on cell apoptosis, proliferation and phenotypic alteration to miR-32-5p upregulation. Furthermore, the overexpression of miR-32-5p could attenuate the influence of DUSP1 on high glucose-induced CFs and collagen synthesis. These findings indicate that the miR-32-5p upregulation might play a necessary pathological role in the diabetic heart.

Various biological processes, such as fibrosis, can be regulated by miRNAs [27, 28]. MiR-32-5p is widely studied in various diseases, including human myeloid leukemia [29], cancer [30], and metabolic syndrome [31]. However, the regulation of miR-32-5p on cardiac fibrosis has not yet been studied, therefore, it was not possible to determine if there was an anomaly in the measuring system or within the samples.

The fibrosis of cardiomyocytes would induce the differentiation of human CFs into myofibroblasts, and would enhance the expression levels of α-SMA and the synthesis of collagen matrix; all of which are necessary events in cardiac remodeling [32]. Our findings observed that the high glucose-induced overexpression of miR-32-5p could enhance the apoptosis rate and induce phenotypic alteration of human CFs, which indicated that the upregulation of miR-32-5p was responsible for the HG-induced proliferation and phenotypic change of human CFs. Moreover, the dominant collagens in ECM including collagen I and collagen III could form fibrils, provide structures in the myocardium, and regulate the proliferation of CFs [33]. Consistent with this, we observed that the overexpression of miR-32-5p induced the expression of collagen I and collagen III.

DUSP1 was reported to dephosphorylate JNK, p38, and ERK in vitro after localizing to the nucleus [34]. Weng et al. observed a decreased expression level of DUSP1 in streptozotocin (STZ)-induced diabetic rats [23]. Moreover, a study by Bueno et al. showed that the decreased expression of DUSP1 could enhance cardiac hypertrophy in the mouse model, which suggests that there is a role of DUSP1 in the pathophysiology of diabetic cardiomyopathy [35]. The observations in our study were in accordance with the findings above. The overexpression of DUSP1 was observed to inhibit the apoptosis rate and phenotypic alteration of human CFs. Additionally, the overexpression of DUSP1 was observed to suppress the expression of collagen I and collagen III. In summary, our findings validate that the downregulation of DUSP1 can aggravate HG-induced cardiac fibrosis.

TGF-β_1_ is crucial for the maintenance and degradation of ECM, and is a main participant in the process of cardiac remodeling [7]. TGF-β_1_ is considered to be a multifunctional cytokine that has biological effects on tissue development and cellular proliferation [36]. Prior studies have proven that the inhibition of TGF-β_1_ can reduce the degree of fibrotic diseases in rats, demonstrating that TGF-β_1_ plays a vital role in fibrosis [36]. Our assay found that the expression level of TGF-β_1_ was notably increased in response to high glucose levels. In addition, the increased expression of TGF-β_1_ was reversed by the overexpression of DUSP1 or by the inhibition of miR-32-5p; the expression was further increased by the overexpression of miR-32-5p. These findings imply that there is a link between DUSP1/miR-32-5p and TGF-β_1_ in diabetic cardiac fibrosis.

Therefore, our results indicate that high glucose-induced cardiac fibrosis is closely associated with the upregulation of miR-32-5p and the downregulation of DUSP1 expression in vitro. In addition, DUSP1 expression in high glucose-induced CFs was downregulated by the upregulation miR-32-5p expression. Furthermore, the regulation of miR-32-5p and DUSP1 in human CFs were confirmed by detecting the expression levels of TGF-β_1_, Collagen I, and Collagen III. Importantly, some researchers have conducted studies on the correlation between miRNA and myocardial fibrosis in vitro and in vivo. Li et al. showed that overexpressing miR-130a in CFs promoted cardiac fibrosis and the in vivo inhibition of miR-130a by anti-miR-130a in mice significantly reduced cardiac fibrosis [37]. Compared with Li et al.’s study, there is no animal models experiment, which is our limitation. But based on reliable cell results, we speculate that our cell model results can be further verified in animals in the future.

## Conclusions

This study has allowed a closer insight into the regulation of miR-32-5p in high glucose-induced cardiac fibrosis. In this study, it was observed that the miR-32-5p was upregulated in human CFs in response to high glucose stimulation and that the overexpression of it could enhance the apoptosis rate and induce the phenotypic alteration of CFs. Furthermore, the directly targeting relationship between miR-32-5p and DUSP1 was demonstrated, and miR-32-5p promoted high glucose-induced cardiac fibroblast proliferation and phenotypic alteration by inhibiting DUSP1.

## Methods

### Cell culture and transient transfection

The human cardiac fibroblasts used in this study were purchased from ScienCell (#6300). The cells were cultured in Fibroblast Medium-2 (FM-2, Cat. #2331) at 37 °C with 95% air and 5% CO_2_ in 0.1% gelatin-coated culture flasks. The culture media contained 5% fetal bovine serum (FBS, GIBCO), 1% penicillin/streptomycin (GIBCO), and 1% fibroblast grown supplement-2 (#2382).

At 70% confluence, the cardiac fibroblasts were transfected with Lipofectamine™ 2000 (Invitrogen) based on the protocol provided by manufacturer. The details are as follows: CFs were seed at a density of 2 × 10^5^ cells per well in 6-well plants. Cells were incubated with a 5% CO_2_ atmosphere at 37 °C until 70% confluence. The cells were transferred to serum-free medium overnight before transfection. Then, miR-32-5p mimics (50 nM) or others treatment group were transfected into CFs using Lipofectamine™ 2000. Two days after transfection, the cells were harvested for the following experiments,.and the transfected cell groups are shown below: (1) NG: control group with a normal concentration of glucose (5.6 mM glucose); (2) OC: osmotic control group with 5.6 mM glucose and 19.4 mM mannitol; (3) HG: test group with a high concentration of glucose (25 mM glucose); (4) mimics group: the cells were transfected with miR-32-5p mimics; (5) inhibitor group: the cells were transfected with a miR-32-5p inhibitor; (6) sh-DUSP1 group: the expression of DUSP1 in CFs was suppressed; (7) DUSP1 group: DUSP1 was overexpressed in CFs.

### Glucose treatment

All human CFs and transfected cells were incubated with a normal concentration (5.6 mM) and a high concentration (25 mM) of glucose for 48 h. As the cells reached 80% confluence, the cells from passage 3–5 were used for the following experiments.

### Flow cytometry

Flow cytometry analysis was performed to identify CFs by examining the expression of the vimentin, fibronectin (FN) and α-actin proteins on cells. The cell suspensions were obtained by using StemPro Accutase cell dissociation buffer (Invitrogen). Then, the cells were washed and fixed with 1.5% phosphate-buffered saline (PBS)/paraformaldehyde (PFA) for 10 min at room temperature. After centrifugation (1500 RCF, 4 °C, 5 min), the cell pellets were resuspended with 500 μL of ice-cold methanol/acetone and were stored in cold room for 30 min to obtain the cell extracts. The extracts were washed with PBS/0.5% BSA (bovine serum albumin) for three times. After which, the cells were incubated with the appropriate primary antibody for 30 min at room temperature as follows: anti-vimentin antibody (ab92547, 1/500 dilution, Abcam), anti-fibronectin antibody (ab2413, 1/200 dilution, Abcam), or anti-alpha smooth muscle actin antibody (ab5694, 1/200 dilution, Abcam). Then, the cells were washed and incubated with a goat anti-rabbit IgG H&L (HRP) secondary antibody (ab6721, 1/1000 dilution, Abcam) at 37 °C for 30 min. The cells were quantified by using BD FACS-Calibur flow cytometer (BD Biosciences) with BD CellQuest™ Pro software according to the manufacturer’s instruction.

### Quantitative real-time polymerase chain reaction (qRT-PCR)

The total mRNA from the cultured CFs was isolated with TRIzol Reagent (Qiagen, USA) and lysed with DNase I (SigmaAldrich) for 30 min following the manufacturer’s instructions. The cDNA was synthesized by using the TaqMan mRNA Reverse Transcription Kit (Applied Biosystems) according to the protocol. The U6 and GAPDH housekeeping genes served as the internal controls. The expression of miR-32-5p and DUSP1 were detected via the SYBR Green kit (Invitrogen). The primer sequences used are shown in Table 1.

**Table 1:** Primers for qRT-PCR

Genes	Sequences
MiR-32-5p	F: 5′-TATTGCACATTACTAAGCCTT-3′
	R: 5′-GAATACCTCGGACCCTGC-3′
DUSP1	F: 5′-GGATACGAAGCGTTTTCGGC-3′
	R: 5′-AGAGGTCGTAATGGGGCTCT-3′
U6	F:5′-GCTTCGGCAGCACATATACTAAAAT-3′
	R: 5′-CGCTTCACGAATTTGCGTGTCAT-3′
GAPDH	F: 5′-GGAAAGCTGTGGCGTGAT-3′
	R: 5′-AAGGTGGAAGAATGGGAGTT-3′

### MTT assay

The proliferation of CFs was analyzed by an MTT assay. MTT reagent (5 mg/mL) (Sigma-Aldrich) was added to the cell culture, and the culture was incubated at 37 °C for 4 h. After this, DMSO was added to dissolve the formazan crystals. The thermomax microplate reader (Bio-TekEL) was used to read the optical density (OD) values at a 595 nm wavelength. Each experiment was performed in triplicate.

### Cell apoptosis

The levels of cell apoptosis were analyzed by flow cytometry using the FITC conjugated Annexin V Apoptosis Detection Kit (BD Biosciences) following the manufacturer’s protocol. The cells in the 6-well plate were lysed with 0.25% trypsin, and then the cell pellets were collected by centrifugation (1500 RCF, 4 °C, 5 min). The cells were then washed twice by using cold PBS together with 1× binding buffer (1 × 10^5^ cells/100 μL binding buffer) and were stained with annexin V-FITC and propidium iodide (PI) for 30 min at room temperature in the dark Each experiment was performed in triplicate.

### Immunofluorescent staining of α-SMA

An immunocytofluorescence staining assay was performed to determine the level of α-SMA protein expression. CFs were added into 96-well plates and were fixed with 4% PFA for 20 min at room temperature. After washing with PBS/5% Tween, the cells were permeabilized with 0.5% TritonX-100 and were blocked with PBS/10% goat serum. Then, the primary antibody anti-alpha smooth muscle actin (ab5694, 1/200 dilution, Abcam) was incubated with the cells for 1 h. After washing, the cells were incubated with the appropriate fluorescence-conjugated secondary antibody (Molecular Probes) for 1 h. The cells were photographed by using the Olympus fluorescence microscope.

### Western blot analysis

The cultured cells were harvested through trypsinization. After being washed with PBS buffer, the cells were resuspended using a lysis buffer (Invitrogen). The protein concentration was determined by a bicinchoninic acid assay via the Pierce BCA Protein Assay Kit (Invitrogen). The proteins content was separated by 10% SDS-PAGE with each lane containing 30 μg of protein sample, and then the separated protein was transferred to a PVDF membrane (Millipore). After blocking with 5% skim milk in a TBS-T solution (20 mM Tris–HCl, pH 7.4, 150 mM NaCl, and 0.1% Tween 20), the membrane was incubated at 4 °C with the following primary antibodies overnight: anti-TGF beta 1 antibody (ab92486, 4 μg/mL, Abcam), anti-collagen I antibody (ab34710, 1/1000 dilution, Abcam), anti-collagen III antibody (ab7778, 1/5000 dilution, Abcam), anti-β actin antibody (ab8226, 1/1000 dilution, Abcam), anti-DUSP1 antibody (ab236501, 1/2000 dilution, Abcam). The membrane was washed with TBS-T, and the membrane was incubated with a goat anti-rabbit IgG H&L (HRP) secondary antibody (ab6721, 1/1000 dilution, Abcam) for 1 h at room temperature. An incubation with an anti-β actin primary antibody was performed as a control. The protein bands were visualized via ECL chemiluminescence (Millipore).

### Dual-luciferase reporter assay

The potential DUSP1 binding site on miR-32-5p was predicted via online database TargetScan 6.2 together with miRanda websites, which shown as 3′-ACGUUA-5′. The wild-type and mutanted regions of DUSP1 were synthesized and inserted into two pET vectors. Before transfection, the CFs were added into 24-well plates and were incubated overnight. Then, either the miR-32-5p mimic or the control mimic and either the DUSP1 3′UTR-WT vector or the DUSP1 3′UTR-mutant vector containing the 3′UTR of DUSP1 gene and the firefly luciferase reporter gene (Promega) were cotransfected using Lipofectamine 2000 (Invitrogen). After 48 h, the luciferase activity was quantified via a dual-luciferase reporter system (Promega) following the standard protocol.

### Statistical analysis

Repeated experiments were performed at least three times for every assay to increase reproducibility. All the data are shown as the mean ± SEM. One-way analysis of variance (ANOVA) was used to analyze the statistical significance. *p *< 0.05 is considered to be statistically significant. All statistical analyses were conducted with GraphPad Prism 6.0 software.

## Additional file

**Additional file 1: Figure S1.** Effect of different concentration of glucose on miR-32-5p and DUSP1 in CFs. (A and B) The RNA expression of DUSP1 and miR-32-5p was detected by a qRT-PCR. (C) The protein expression of DUSP1 was measured by a western blot assay. The data are presented as the mean ± SEM (n = 3). *p < 0.05 (compared with the 5.6 mM group).

## Data Availability

All data generated or analysed during this study are included in this published article.

## Abbreviations

ANOVA: analysis of variance
CFs: cardiac fibroblasts
DM: diabetes mellitus
DUSP: dual-specificity protein phosphatase
ECM: extracellular matrix
ERK: extracellular signal-regulated kinase
FN: fibronectin
HG: high glucose
JNK: c-Jun N-terminal kinase
MAPKs: mitogen-activated protein kinase
PBS: phosphate buffered saline
PFA: paraformaldehyde
TGF-β: transforming growth factor-β
