# Supplementary figures and images for: MiR-32-5p influences high glucose-induced cardiac fibroblast proliferation and phenotypic alteration by inhibiting DUSP1
Source: BMC Mol Biol. 2019 Aug 22;20:21. doi: 10.1186/s12867-019-0135-x (PMC6704591; doi:10.1186/s12867-019-0135-x)

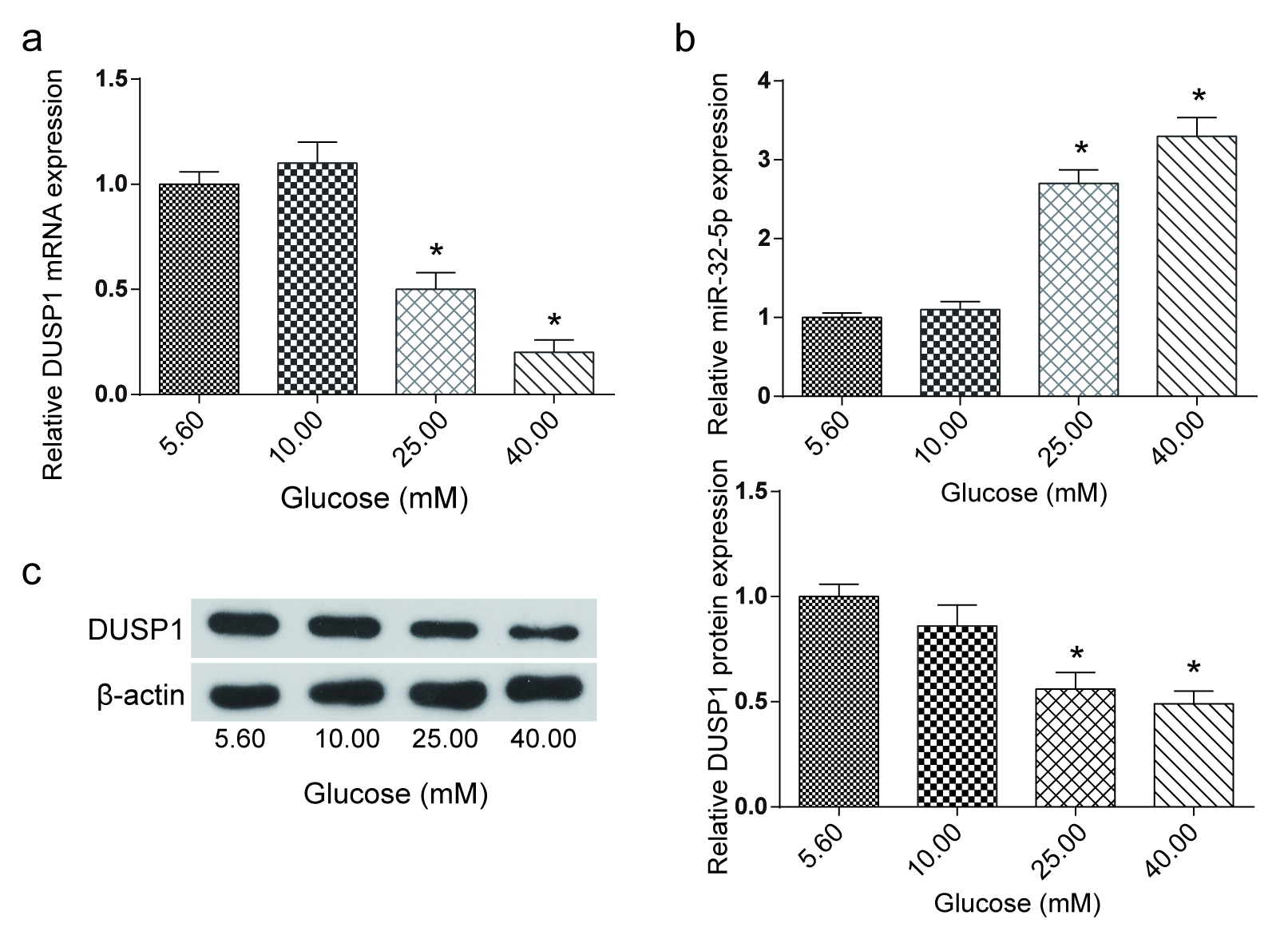

Supplement: Supplementary file 1 — Additional file 1: Figure S1. Effect of different concentration of glucose on miR-32-5p and DUSP1 in CFs. (A and B) The RNA expression of DUSP1 and miR-32-5p was detected by a qRT-PCR. (C) The protein expression of DUSP1 was measured by a western blot assay. The data are presented as the mean ± SEM (n = 3). *p < 0.05 (compared with the 5.6 mM group). [file 12867_2019_135_MOESM1_ESM.jpg]
